# Biogenic synthesis of gold nanoparticles mediated by *Spondias dulcis* (Anacardiaceae) peel extract and its cytotoxic activity in human breast cancer cell

**DOI:** 10.1016/j.toxrep.2022.04.031

**Published:** 2022-05-11

**Authors:** Chiravoot Pechyen, Khanittha Ponsanti, Benchamaporn Tangnorawich, Nipaporn Ngernyuang

**Affiliations:** a Department of Materials and Textile Technology, Faculty of Science and Technology, Thammasat University, Pathum Thani 12120, Thailand; b Department of Physics, Faculty of Science and Technology, Thammasat University, Pathum Thani 12120, Thailand; c Thammasat University Center of Excellence in Modern Technology and Advanced Manufacturing for Medical Innovation, Thammasat University, Pathum Thani 12120, Thailand; d Chulabhorn International College of Medicine, Thammasat University, Pathum Thani 12120, Thailand

**Keywords:** Gold nanoparticles, Green synthesis, Phytosynthesis, Anticancer, Cytotoxicity, Human breast cancer cells

## Abstract

Green synthesis is a new paradigm for the preparation of gold nanoparticles (AuNPs) due to its cost-effectiveness and favorable environmental impact. This study presented a simple phytosynthesis process for the preparation of AuNPs utilizing the aqueous peel extract of *Spondias dulcis* (SPE) (Anacardiaceae) as both a reducing and stabilizing agent. A visual color change from yellow to purple during the reaction implied the successful formation of SPE-AuNPs, which was confirmed by UV–vis spectroscopy. Transmission electron microscopy (TEM) images indicated that the SPE-AuNPs were predominantly spherical with a mean size of 36.75 ± 11.36 nm, and were comprised of crystalline Au, as indicated by X-ray diffraction. In terms of their potential application, SPE-AuNPs exhibit significant cytotoxic activity in a dose- and time-dependent manner to MCF-7 human breast cancer cells, while being non-toxic to Vero normal cells. The treatment of MCF-7 cells with SPE-AuNPs increased the production of intracellular reactive oxygen species (ROS). Herein, the findings highlight the potential contribution of phytosynthesized SPE-AuNPs to the development of novel nanomedicines for cancer treatment.

## Highlights

• SPE-AuNPs were synthesized by aqueous extract of *Spondias dulcis* peel.
• SPE-AuNPs were spherical with average size of 36.75 ± 11.36 nm.
• SPE-AuNPs were showed significant cytotoxic effects against MCF-7 breast cancer cell in dose- and time-dependent manner.
• SPE-AuNPs induce the formation of ROS, triggering mitochondrial and nuclear damage.

## Graphical Abstract

**Figure:**
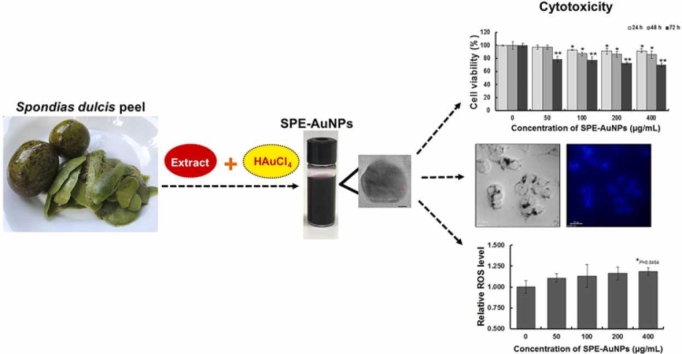

## 1 Introduction

Cancer is a major global health issue, accounting for nearly 10 million deaths worldwide in 2020 [1]. Cancer treatments can be prolonged and difficult, taking the forms of surgery, radiotherapy, immunotherapy, hormonal therapy, and chemotherapy. Apart from surgery, however, these treatments can affect healthy tissue in addition to the tumors, resulting in side effects. Drug resistance and toxicity add to the complex nature of cancer treatment [2]. Consequently, the development of more effective chemotherapeutic agents is an active area of pharmaceutical research.

The advent of nanotechnology and the application of nanomaterials in medicine has the potential to greatly impact human health through the prevention, diagnosis, and treatment of diseases [3]. Due to their high surface area and nanoscale size (< 100 nm) [4], nanoparticles (NPs) are attracting considerable attention, with gold nanoparticles (AuNPs) exhibiting the most potential amongst metal nanoparticles in medicinal applications (cancer diagnosis and therapy, drug delivery, gene therapy, biomarker mapping, targeted therapy, and molecular imaging) due to their high stability [5], [6], [7], [8], [9], [10], [11], [12], [13]. Three strategies can be employed to synthesize AuNPs: physical, chemical, and biological [14]. Conventional physical and chemical methods tend to be energy-intensive [15], with chemical processes particularly requiring solvent use, which contributes to the environmental impact as well as overall cost [16], [17]. In contrast, biological or green syntheses are designed to be inexpensive and eco-friendly, using agents obtained from natural sources (plants [18], bacteria [19], algae [20], or fungi [21]) for the production of AuNPs. Phytosynthesis, which utilizes plant biomasses or extracts that serve to act as reducing agents for AuNPs, is of particular interest [22], [23]. According to previous studies, various parts of plants such as roots, stems, leaves, flowers, bark, latex, fruits, and seeds can be used for metal AuNP synthesis. Every part of the plant has been proven to be useful, especially the leaves [24], [25], [26], [27]. However, few reports have focused on fruit peels [28], [29], [30].

*Spondias dulcis* (Anacardiaceae) is a tropical tree commonly found in tropical, subtropical, and temperate climate zones. *S. dulcis* is widely spread and has been used in food and as traditional medicine. *S. dulcis* produces fruits that are called Otaheite apples or Golden apples [31]. The mature-green fruits are also consumed raw, and the ripe fruits are used to produce juice and jam beverages. The fruit is used to cure sore throat, internal ulceration, itchiness, and inflammation of the skin, as well as to enhance sight, treat eye infections, and perform as an antidote [32]. Moreover, the pulp of the fruit contains compounds that exhibit potent antimicrobial, antioxidant, cytotoxic, and thrombolytic activity [33]. Despite this, no study has focused on the biological compounds and applications of the peel of *S. dulcis*.

For this reason, the present study aims to highlight the potential of *S. dulcis* peel extract in the synthesis of AuNPs for use in anticancer therapeutics. To the best of the authors’ knowledge, there are no previous reports in the literature concerning biosynthesized AuNPs using *S. dulcis* peel extracts. Thus, biosynthesized AuNPs were thoroughly characterized in the present study, and their *in vitro* cytotoxicity was evaluated against both Vero normal cell and the MCF-7 human breast cancer cell line.

## 2 Experimental

### 2.1 Materials

#### 2.1.1 Preparation of S. dulcis peel extract (SPE)

Fresh fruits of *S. dulcis* were collected in August 2020, from Pathum Thani, Thailand. *S. dulcis* fruits were washed thoroughly with tap water prior to peeling. The peels were collected and then cut into small pieces. For extraction, a total of 20 g of *S. dulcis* peel was boiled in 100 mL of deionized (DI) water for 1 hr under constant stirring, after which it was left to cool, and then filtered through Whatman filter paper (No. 1). The aqueous SPE was stored at 4 °C for use in AuNPs synthesis.

### 2.2 Phytochemical analysis

#### 2.2.1 Total phenolic content (TPC)

The evaluation of TPC in SPE was determined using the Folin-Ciocalteu assay [34]. Briefly, 25 μL of SPE extract was mixed with 25 μL of Folin-Ciocalteu reagent in a 96-well plate and incubated for 3 min. After an incubation period, 125 μL of 20% sodium carbonate (Na_2_CO_3_) was added to the solution mixture. The solution mixture was then incubated at 40 °C for 20 min in the dark and its absorbance was measured at 685 nm by using a microplate reader. A calibration curve was prepared using catechin as standard, and the TPC was expressed as milligrams of catechin equivalent (CAE) per gram of sample in the extract (mg CAE/g). The procedure was repeated in triplicate.

#### 2.2.2 Total flavonoid content (TFC)

The TFC of SPE was measured by the aluminum chloride (AlCl_3_) colorimetric method [35]. Briefly, 100 μL of SPE extract was added to 100 μL of 2% AlCl_3_ solution and then incubated at room temperature for 10 min. After an incubation period, the absorbance intensity was measured at 415 nm by using a microplate reader. The TFC was expressed as milligrams of quercetin equivalent (QE) per gram of the sample in the extract (mg QE/g) using the linear equation based on the standard calibration curve.

### 2.3 Synthesis of AuNPs using SPE

Gold (III) chloride trihydrate (99.9%, HAuCl_4_.3 H_2_O, Sigma-Aldrich) was used as the gold precursor. AuNPs were synthesized by adding 1% of aqueous SPE to 1 mM aqueous gold (III) chloride solution at room temperature [36]. The reaction was then stirred continuously for 1 hr, with the formation of AuNPs being observed by a color change from yellow to purple. After centrifugation at 10,000 rpm for 30 min to remove excess reagents, the resulting pellets were dispersed in 1 mL of sterile, double-distilled water. Thereafter, the centrifugation and re-dispersing processes were repeated three times. Subsequently, the purified pellets of synthesized AuNPs (SPE-AuNPs) were dried at 45 °C in a vacuum oven before characterization and use.

### 2.4 Characterization of synthesized AuNPs

UV–vis spectra of AuNPs dispersions were recorded over a 400–700 nm range using a Varioskan Flash spectrophotometer (Thermo Scientific, USA). The size and morphology of the synthesized AuNPs were investigated using transmission electron microscopy (TEM) (JEOL, JEM-2100 Plus, JAPAN). For TEM, samples were dispersed in deionized water and dropped on a carbon-coated copper grid. Images were obtained using an accelerating voltage of 120 kV. The surface charge of AuNPs was probed through zeta potential measurements (ZetaSizer Nano ZS, Malvern Instruments Ltd., UK). X-ray diffraction (XRD) patterns of the AuNPs were obtained using a Bruker AXS D8 Discover powder diffractometer, with SPE rating using the following parameters: voltage 40 kV, current 30 mA, CuKα radiation (1.5406 Å), 2θ range 30–90°, and scan rate 2°/min. To identify functional groups in SPE and on the surface of SPE-AuNPs, Fourier transform infrared spectrophotometry (FTIR) studies were undertaken over a 4000–400 cm^−1^ range (Nicolet iS50, Thermo Scientific, USA) with samples being prepared as KBr disks. Thermogravimetric analyses (TGA) were carried out with a heating rate of 10 °C/min using a Perkin Elmer Diamond TG/DTA Perkin Elmer TG/DTA system.

### 2.5 Cell culture

Vero normal African green monkey kidney epithelial cells (ATCC® CCL-81™) and MCF-7 human breast cancer cells (ATCC® HTB-22™) were obtained from the American Type Culture Collection (ATCC; Manassas, USA). The cells were grown in Dulbecco’s Modified Eagle Medium (DMEM) supplemented with 10% v/v fetal bovine serum and 1% v/v penicillin/streptomycin (P/S) (all from Gibco, USA). All cultures were grown at 37 °C in a humidified incubator with 5% CO_2_.

### 2.6 Cell cytotoxicity evaluations

The cytotoxicity of SPE-AuNPs was evaluated using the Celltiter 96® aqueous one solution cell proliferation assay (MTS assay; Promega, USA) according to the manufacturer’s guidelines. In this protocol, cells were plated into 96-well culture plates and exposed to various concentrations (50, 100, 200, and 400 μg/mL) of SPE-AuNPs. Untreated cells served as the control group. All cultures were incubated at 37 °C with 5% CO_2_ in a humid atmosphere for 24, 48, and 72 hrs. At the end of the incubation period, the medium was removed, and the cells were washed with 100 μL of phosphate-buffered saline (PBS). The supernatant was replaced with 100 μL of DMEM containing 20 μL of MTS solution, and then cells were incubated for 2 hrs, after which the supernatant was transferred to a new 96-well plate to prevent interference from absorbed SPE-AuNPs. The absorbance was measured at 495 nm using a microplate reader. Experiments were conducted in triplicate, and the percentages of viable cells were calculated with respect to control (untreated cells).

### 2.7 Cell staining

The degree of cellular apoptosis was quantified by nuclear morphology, as visualized by treatment with a Hoechst 33258 solution (5 μg/mL) (Sigma-Aldrich, USA), a fluorescent DNA-binding dye. Cells were seeded into a 6-well plate and treated with different concentrations of SPE-AuNPs for 72 hrs. After treatment, the cells were fixed with cold methanol for 10 min and then washed twice with PBS. The cells were then stained with the Hoechst 33258 solution and incubated at room temperature for 10 min. Changes in nuclear morphology were observed using a fluorescence microscope. For the mitochondrial transmembrane potential assay (rhodamine 123 staining), the Hoechst 33258-stained cells were stained with rhodamine 123 (2.5 μg/mL in PBS) and incubated for 15 min at room temperature in the dark. After three washes with PBS, the cells were dried thoroughly and observed under a fluorescence microscope.

### 2.8 Reactive oxygen species (ROS) assay

Intracellular ROS generation was investigated using the reagent 2′, 7′-dichlorofluorescein diacetate (DCFDA). MCF-7 cells were treated with a set concentration of SPE-AuNPs (0–400 μg/mL) and then incubated for 48 h. After incubation, the media was removed and cells were washed with phosphate-buffered saline (PBS). Subsequently, DCFH-DA (10 μM) was added to the cells and incubated for another 30 min in the dark at 37 °C. The fluorescence intensity was measured at 485 nm (excitation) and 535 nm (emission) using a microplate reader.

### 2.9 Statistical analysis

The statistical significance of data was determined by analysis using one-way ANOVA. All data are presented as mean ± standard deviation (SD). Data were considered statistically significant at p < 0.05.

## 3 Results and discussion

### 3.1 Phytochemical analysis

The Folin-Ciocalteu method was applied for the determination of TPC in the fruit peel of *S. dulcis*. Calibration curves were prepared using catechin (CA) as standard. The TPC of the SPE was calculated from the calibration curve using regression equation Y = 0.1737X - 0.1606, R² = 0.9935 followed by the formula C = cV/m, and expressed as milligrams per gram of the extract (mg CAE/g extract). The TPC of the SPE was 10.27 ± 0.46 mg CAE/g extract.

The TFC of the SPE was estimated by AlCl_3_ assay using quercetin as standard. The average absorbance values obtained at different concentrations of quercetin were used to plot the calibration curve. The result was derived from the calibration curve using regression equation Y = 0.3021X - 0.2722, R^2^ = 0.995 and expressed as quercetin (QE) equivalents per gram of sample in weight (mg/g). The TFC value of SPE was 12.67 ± 0.32 mg QE/g.

Our results suggest that the *S. dulcis* peel extract is composed of phenolic compounds and flavonoids, similar to the findings shown in a previous study [37]. Moreover, several studies have suggested that these bioactive compounds are the main reducing agents involved in the synthesis of AuNPs [38].

### 3.2 Characterization of *S. dulcis* peel extract synthesized AuNPs

**Fig. 1:**
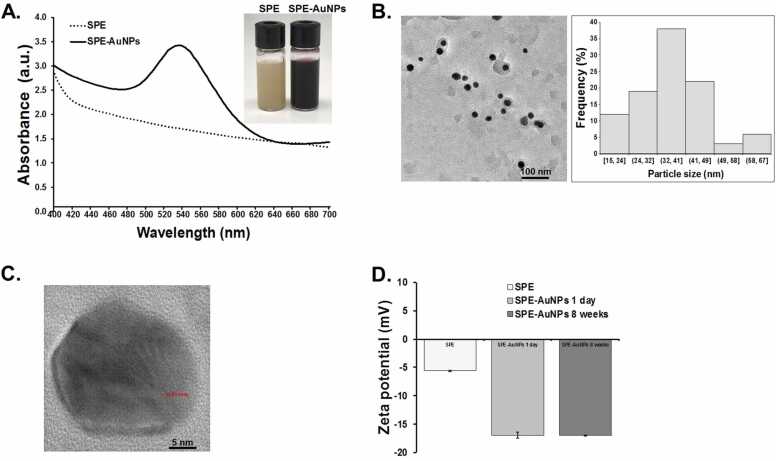
Characterization of SPE-AuNPs. (A.) UV–vis absorption spectra of SPE-AuNPs showing the λ_max_ at 536 nm., (B.) TEM image of SPE-AuNPs (left) with a histogram plot of the distribution of sizes (right)., (C.) HRTEM image of SPE-AuNPs with lattice fringes pattern., and (D.) Zeta potential value for SPE, SPE-AuNPs 1 day, and SPE-AuNPs 8 weeks.

Initial indications that *S. dulcis* peel extract was an effective agent for AuNPs synthesis came from the visual color changes of the Au (III). The color change from yellow to purple was explored further using UV–vis spectroscopy, and absorbance was observed at a λ_max_ of 536 nm (Fig. 1A), consistent with the surface plasmon resonance (SPR) excitation peak in AuNPs [39]. The peak is a distinguishing feature of 30–50 nm diameter spherical AuNPs [40], [41].

The size and morphology of the obtained SPE-AuNPs were investigated using TEM imaging, as shown in Fig. 1B. It was observed that the SPE-AuNPs were well dispersed and mostly spherical with an average particle diameter of 36.75 ± 11.36 nm. HRTEM images (Fig. 1C) exhibited clear lattice fringes of 0.211 nm, which demonstrates the AuNPs occurred preferentially in the (111) direction [42]. This was further confirmed by XRD analysis.

Zeta potential values are widely used as indicators of the stability of colloidal dispersions. The absolute value is related to the net surface charge on the external surface of particles. Nanoparticles with zeta potentials of less than − 30 mV or more than 30 mV are normally considered stable [43]. However, the SPE-AuNPs exhibit a low zeta potential value with a negative surface charge of − 16.93 ± 0.51 mV (Fig. 1D), indicating that they might be less stable and thus have a higher tendency to aggregate and form larger particles. Solutions of SPE-AuNPs were preserved in a sealed vial at room temperature to observe the stability of the colloid solution. The colloidal solution of SPE-AuNPs was found to be stable for over 8 weeks, and no gold particles settled to the bottom (not aggregate). Moreover, the zeta potential of the colloidal solution of SPE-AuNPs was measured again after 8 weeks, with retained constant zeta potential (−16.96 ± 0.11 mV) suggesting that SPE-AuNPs exhibit an acceptable degree of stability.

**Fig. 2:**
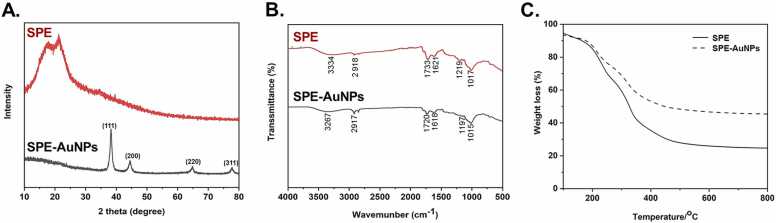
Powder XRD patterns, FTIR spectra, and TGA analysis of SPE and SPE-AuNPs., (A.) Powder XRD patterns, (B.) FTIR spectra of SPE (red) and SPE-AuNPs (black), and (C.) TGA of SPE-AuNPs prepared using *S. dulcis* peel extract.

The crystalline nature of SPE-AuNPs was confirmed by XRD analysis. The XRD patterns are shown in Fig. 2A. SPE-AuNPs exhibited four distinct peaks at approximately 38.3°, 44.5°, 64.8°, and 77.7°, which relate to reflections from the (1 1 1), (2 0 0), (2 2 0), and (3 1 1) planes of face-centered cubic (FCC) Au, respectively. These peaks agree with standard report values from JCPDS (JCPDS card no. 04-0784), confirming that biogenic AuNPs synthesized by SPE are composed of elemental gold [44], [45].

FTIR analysis was performed to identify the functional groups involved in the reduction of gold ions and the stabilization of SPE-AuNPs (Fig. 2B). The FTIR spectra of both SPE and SPE-AuNPs were recorded. The FTIR spectra of SPE exhibited characteristic peaks at 3334, 2918, 1733, 1621, 1219, and 1017 cm^−1^, respectively. The peak at 3334 cm^−1^ could be attributed to the stretching vibrations of O-H [46]. The peak at 2918 cm^−1^ was assigned to the symmetric and asymmetric C-H stretching vibrations of the methyl, methylene, and methoxy groups [47]. The peaks at 1733 and 1621 cm^−1^ were assigned to the C=O stretching in the aldehyde group and C=C stretching in aromatic compounds, respectively, while the peaks at 1219 and 1017 cm^−1^ were assigned to the stretching vibrations of the C-O stretching carboxylic acid group and C-N aromatic and aliphatic amines, respectively [48], [49], [50], [51]. These bands shift in the spectra of SPE-AuNPs to 3267, 2917, 1720, 1618, 1197, and 1082 cm^−1^. This suggests that the carboxyl, hydroxyl, and amide groups in SPE could be attributed to the reduction and stabilization process during AuNPs synthesis. Analogous results were reported in previous studies [28], [29], [30].

The thermal behavior and thermal stability of SPE-AuNPs were investigated using the TGA method. The thermal stability of SPE is lower than that of SPE-AuNPs (Fig. 2C). The first weight-loss stage from room temperature to 250 °C refers to the evaporation of adsorbed water. The second prominent decrease in weight is from 250 ºC to 400 °C, which is due to the loss of organic compounds. The SPE rapidly lost about 50% of weight in a temperature region below 350 ºC. In contrast, the curve of SPE-AuNPs showed 50% weight loss in a high-temperature region of approximately 470 ºC, though there was no weight loss in a higher temperature region. The lack of weight loss by SPE-AuNPs in a high-temperature region indicated the protective effect of the organic compounds stabilizing AuNPs [52].

### 3.3 Cytotoxicity of SPE-AuNPs

**Fig. 3:**
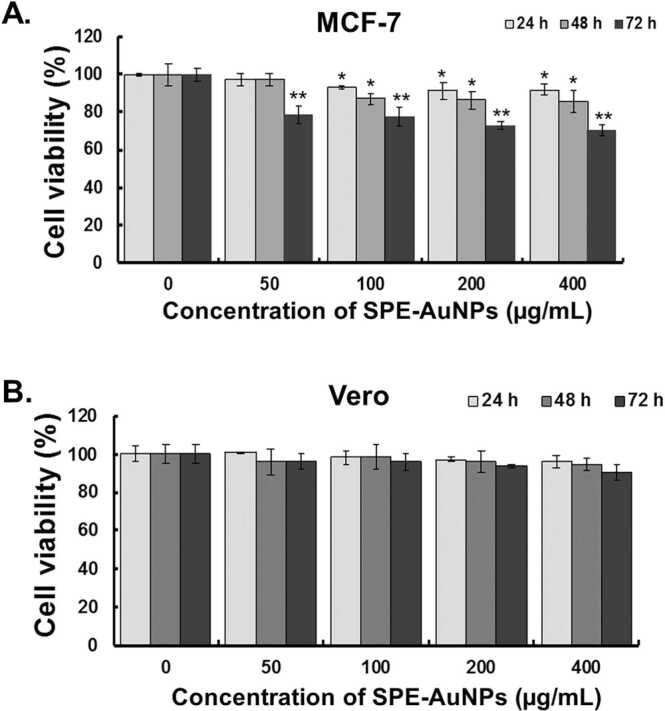
Cytotoxic effects of SPE-AuNPs on different cell types. MCF-7 human breast cancer cells (A) and Vero normal cells (B) were exposed to different concentrations of SPE-AuNPs for 24, 48 and 72 h, and the effect on cell viability analyzed by MTS assay. The number of viable cells after treatment is expressed as a percentage of the vehicle only control (untreated cells). Experiments were done in triplicate and the bars in the graph represent SD (*P < 0.05, **P < 0.01).

In this study, the toxic effects of SPE-AuNPs on Vero normal cells and MCF-7 breast cancer cells were investigated using the MTS assay. Cell viability was screened for different SPE-AuNPs concentrations (50, 100, 200, and 400 μg/mL), and gold nanoparticles inhibited MCF-7 cell viability in a dose- and time-dependent manner when compared to untreated cells (control), as shown in Fig. 3A. Vero cells were used to mimic normal human cells in this study. Interestingly, SPE-AuNPs were found to be less cytotoxic against these cells, even at a high concentration (400 μg/mL) (Fig. 3B). Cytotoxicity is dependent on many factors including the type of cancer cell, and the characteristics of the nanoparticles such as size, shape, and surface chemistry [53]. The enhanced cytotoxicity of AuNPs against cancer cells is thought to be due to their higher uptake capacity as a consequence of metabolic anomalies and rapid proliferation rate [54], [55]. When summarized, these results suggest that SPE-AuNPs inhibit the growth of MCF-7 cells in a dose- and time-dependent manner, and this effect is most prominent 72 hrs post-treatment.

**Fig. 4:**
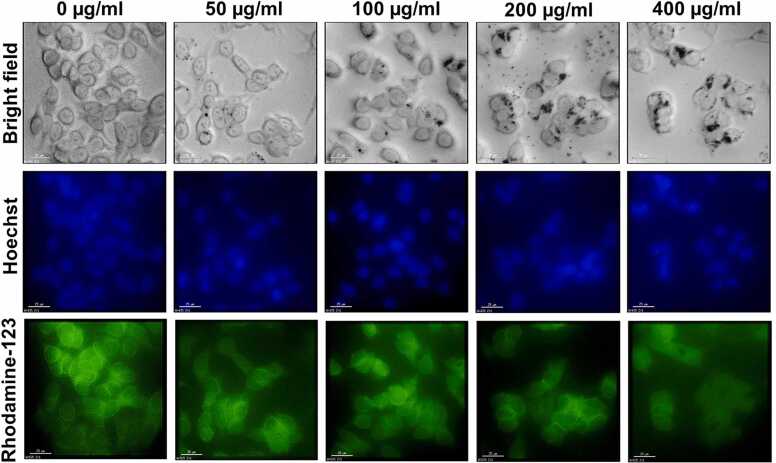
The effect of SPE-AuNPs on cellular apoptosis. Bright field (top row) and fluorescence images of MCF-7 cells treated with different concentrations of SPE-AuNPs after 72 h incubation. Fluorescence images were captured post-staining of cells with Hoechst 33258 and Rhodamine 123 (middle and bottom row, respectively).

To further confirm the cytotoxic effects of SPE-AuNPs on cell morphology, Hoechst 33258 stained cells were observed under a fluorescent microscope. In the early stages of cellular apoptosis, cell shrinkage and pyknosis are visible using light microscopy [56]. As shown in Fig. 4, untreated MCF-7 cells exhibit uniform sizes and regular shapes. In contrast, cells treated with SPE-AuNPs, in a dose-dependent form of response, lose their cellular shape (cell shrinkage and chromatin condensation) and exhibit a decrease in cellular confluency. These results highlight the potential cytotoxic effects of SPE-AuNPs in MCF-7 breast cancer cells. Several reports have detailed the anticancer activity of biogenic AuNPs synthesized from different fruit peel extracts. *Musa paradisiaca* peel extract synthesized AuNPs showed anticancer activity against human lung cancer (A549) cells at higher concentrations of 100 mg/mL [57]. Similarly, *Annona muricata* peel extract synthesized AuNPs exhibited cytotoxic activity on Hep2 liver cancer cells while remaining non-toxic to Vero cells [58]. Based on these results, it is evident that biosynthesized SPE-AuNPs are cytotoxic to cancer cells and could be used as anticancer agents or drug delivery systems in the future.

SPE-AuNPs induced the loss of mitochondrial membrane potential in MCF-7 cells as determined by Rhodamine-123 (lipophilic cationic dye). The loss of mitochondrial membrane potential results in reduced green fluorescence intensity. In this experiment, it was observed that the uptake of Rhodamine-123 was substantially lower than that of untreated MCF-7 cells (Fig. 4). Decreased green fluorescence in SPE-AuNPs treated cells indicated mitochondrial dysfunction, which was thought to be an early stage of apoptosis [59]. Similarly, several studies have reported that AuNPs can induce cell death and apoptosis via cytotoxic stress, and are also responsible for mitochondrial membrane disruption [60], [61].

### 3.4 SPE-AuNPs induce ROS formation

**Fig. 5:**
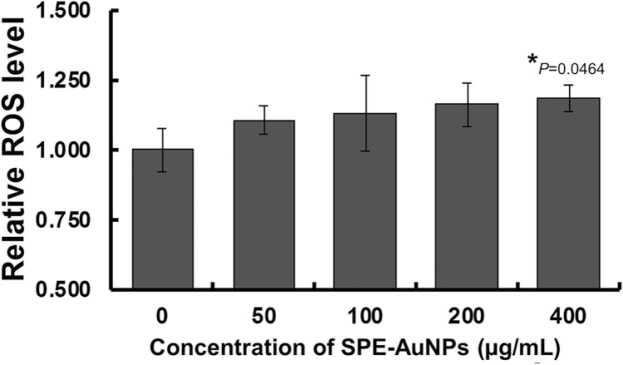
The effect of SPE-AuNPs on ROS production in MCF-7 breast cancer cells. Each bar represents mean ± SD of three independent observations. *P < 0.05 is considered as being statistically significant.

Several studies have reported the ability of AuNPs to induce the formation of reactive oxygen species (ROS) [58], [62]. In this work, MCF-7 cells were exposed to SPE-AuNPs for 72 hrs, and cellular ROS levels were then examined using DCFH-DA staining. The results in Fig. 5 demonstrate that ROS production levels are significantly higher in SPE-AuNPs treated cells compared to the controls in a dose-dependent manner (Fig. 5, *P* = 0.0464), which indicates that SPE-AuNPs induce oxidative cell damage through ROS generation. These results are consistent with past works that highlighted green-synthesized AuNPs induced effects on DNA oxidative damage, suggesting that AuNPs mediate ROS-induced genotoxicity, which may be a basis for their cytotoxic effects [63], [64], [65]. Several herbal extractions including *Marsdenia tenacissima* [63] and *Scutellaria barbata* [64] have been used in the green synthesis of AuNPs and induced toxicity in cancer cells. Interestingly, Parida and colleagues [65] conducted extensive studies on the exposure of *Syzygium aromaticum*-mediated AuNPs in human lymphoma cell line (SUDHL-4) cells. Their results showed cytotoxic activity by the activation of ROS production, dysfunction of mitochondria, and caspase-dependent apoptosis. A previous report concluded that ROS signaling may play an important role in AuNP-induced apoptotic cell death in SUDHL-4 cells [65].

## 4 Conclusions

Green synthesis is a viable strategy for the rapid, cost-effective, and environmentally friendly preparation of gold nanoparticles. In this study, AuNPs were synthesized using *S. dulcis* peel extract, which contains biomolecules for the reduction of Au (III) and stabilization of the resultant SPE-AuNPs. The SPE-AuNPs were fully characterized via a range of techniques and were proven to be selective in terms of their cytotoxicity to MCF-7 cancer cells relative to normal controls. While further experiments indicated that SPE-AuNPs induce ROS production, the exact mechanism behind the in vitro and in vivo anticancer properties of SPE-AuNPs remains unclear and requires additional investigation.

## CRediT authorship contribution statement

**Chiravoot Pechyen:** Conceptualization, Visualization, Investigation. **Khanittha Ponsanti:** Methodology, Data curation. **Benchamaporn Tangnorawich:** Visualization, Investigation. **Nipaporn Ngernyuang:** Conceptualization, Methodology, Data curation, Visualization, Investigation, Writing – review & editing.

## Declaration of Competing Interest

The authors declare that they have no known competing financial interests or personal relationships that could have appeared to influence the work reported in this paper.

## References

[1] Sung H., Ferlay J., Siegel R.L., Laversanne M., Soerjomataram I., Jemal A., Bray F. (2021). Global cancer statistics 2020: GLOBOCAN estimates of incidence and mortality worldwide for 36 Cancers in 185 countries. CA Cancer J. Clin..

[2] Baskar R., Lee K.A., Yeo R., Yeoh K.W. (2012). Cancer and radiation therapy: current advances and future directions. Int. J. Med. Sci..

[3] El-Sayed A., Kamel M. (2020). Advances in nanomedical applications: diagnostic, therapeutic, immunization, and vaccine production. Environ. Sci. Pollut. Res..

[4] Laurent S., Forge D., Port M., Roch A., Robic C., Elst V.L., Muller R.N. (2010). Magnetic iron oxide nanoparticles: synthesis, stabilization, vectorization, physicochemical characterizations, and biological applications. Chem. Rev..

[5] El-Sayed I.H., Huang X., El-Sayed M.A. (2006). Selective laser photo-thermal therapy of epithelial carcinoma using anti-EGFR antibody conjugated gold nanoparticles. Cancer Lett..

[6] Huang X., Jain P.K., El-Sayed I.H., El-Sayed M.A. (2008). Plasmonic photothermal therapy (PPTT) using gold nanoparticles. Lasers Med. Sci..

[7] Ghosh P., Han G., De M., Kim C.K., Rotello V.M. (2008). Gold nanoparticles in delivery applications. Adv. Drug. Deliv. Rev..

[8] Han G., Ghosh P., Rotello V.M. (2007). Functionalized gold nanoparticles for drug delivery. Nanomedicine.

[9] Cheng Y., Samia C., Meyers J.D., Panagopoulos I., Fei B., Burda C. (2008). Highly efficient drug delivery with gold nanoparticle vectors for in vivo photodynamic therapy of cancer. J. Am. Chem. Soc..

[10] Rosi N.L., Giljohann D.A., Thaxton C.S., Lytton-Jean A.K., Han M.S., Mirkin C.A. (2006). Oligonucleotide-modified gold nanoparticles for intracellular gene regulation. Science.

[11] Tkachenko A.G., Xie H., Coleman D., Glomm W., Ryan J., Anderson M.F., Franzen S., Feldheim D.L. (2003). Multifunctional gold nanoparticle-peptide complexes for nuclear targeting. J. Am. Chem. Soc..

[12] Pissuwan D., Niidome T., Cortie M.B. (2011). The forthcoming applications of gold nanoparticles in drug and gene delivery systems. J. Control Release.

[13] Qu X., Li Y., Li L., Wang Y., Liang J., Liang J. (2015). Fluorescent gold nanoclusters: synthesis and recent biological application. J. Nanomater.

[14] Yeh Y.C., Creran B., Rotello V.M. (2012). Gold nanoparticles: preparation, properties, and applications in bionanotechnology. Nanoscale.

[15] Freitas L.F., Varca G.H.C., Batista J.G.S., Lugão A.B. (2018). An overview of the synthesis of gold nanoparticles using radiation technologies. Nanomater.

[16] Parashar U.K., Saxena P.S., Srivastava A. (2009). Bioinspired synthesis of silver nanoparticles. Dig. J. Nanomater. Biostruct..

[17] Parashar V., Parashar R., Sharma B., Pandey A.C. (2009). Partenium leaf extract mediated synthesis of silver nanoparticles: a novel approach towards weed utilization. Dig. J. Nanomater. Biostruct..

[18] Santhoshkumar J., Rajeshkumar S., Kumar V.S. (2017). Phyto-assisted synthesis, characterization and applications of gold nanoparticles - a review. Biochem. Biophys. Rep..

[19] Kumar K.S., Kumar G., Prokhorov E., Luna-Bárcenas G., Buitron G., Khanna V.G., Sanchez I.C. (2014). Exploitation of anaerobic enriched mixed bacteria (AEMB) for the silver and gold nanoparticles synthesis. Colloids Surf. A Physicochem. Eng. Asp..

[20] Costa L.H., Hemmer J., Wanderlind E.H., Gerlach O.M.S., Santos A.L.H., Tamanaha M.S., Bella-Cruz A., Corrêa R., Bazani H.A.G., Radetski C.M., Almerindo G.I. (2020). Green synthesis of gold nanoparticles bbtained from Algae *Sargassum cymosum*: optimization, characterization and stability. Bio. Nano Sci..

[21] Mishra A., Tripathy S.K., Yun S. (2012). Fungus mediated synthesis of gold nanoparticles and their conjugation with genomic DNA isolated from *Escherichia coli* and *Staphylococcus aureus*. Process Biochem..

[22] Noruzi M. (2015). Biosynthesis of gold nanoparticles using plant extracts. Bioprocess Biosyst. Eng..

[23] Mohammadinejad R., Karimi S., Iravani S., Varma R.S. (2016). Plant-derived nanostructures: types and applications. Green Chem..

[24] Philip D. (2010). Rapid green synthesis of spherical gold nanoparticles using *Mangifera indica* leaf. Spectrochim. Acta Part A.

[25] Dash S.S., Bag B.G., Hota P. (2015). *Lantana camara* Linn leaf extract mediated green synthesis of gold nanoparticles and study of its catalytic activity. Appl. Nanosci..

[26] Kumar V.G., Gokavarapu S.D., Rajeswarietal A. (2011). Facile green synthesis of gold nanoparticles using leaf extract of antidiabetic potent *Cassia auriculata*. Colloids Surf. B. Biointerfaces.

[27] Sadeghi B., Mohammadzadeh M., Babakhani B. (2015). Green synthesis of gold nanoparticles using *Stevia rebaudiana* leaf extracts: characterization and their stability. J. Photochem. Photobiol. B Biol..

[28] Yang N., Weihong L., Hao L. (2014). Biosynthesis of Au nanoparticles using agricultural waste mango peel extract and its in vitro cytotoxic effect on two normal cells. Mater. Lett..

[29] Yang B., Qi F., Tan J., Yu T., Qu C. (2019). Study of green synthesis of ultrasmall gold nanoparticles using *Citrus sinensis* peel. Appl. Sci..

[30] Gangapuram B.R., Bandi R., Alle M., Dadigala R., Kotu G.M., Guttena V. (2018). Microwave assisted rapid green synthesis of gold nanoparticles using *Annona squamosa* L peel extract for the efficient catalytic reduction of organic pollutants. J. Mol. Struct..

[31] Mitchell J.D., Daly D.C. (2015). A revision of *Spondias* L. (Anacardiaceae) in the neotropics. PhytoKeys.

[32] Rahmatullah M., Ferdausi D., Mollik M.A., Azam M.N., Rahman M.T., Jahan R. (2009). Ethnomedical survey of bheramara area in Kushtia district, Bangladesh. Am.-Eurasian J. Sustain. Agric..

[33] Islam S.A., Ahmed K.T., Manik M.K., Wahid A., Kamal C.H.I. (2013). A comparative study of the antioxidant, antimicrobial, cytotoxic and thrombolytic potential of the fruits and leaves of *Spondias dulcis*. Asian Pac. J. Trop. Biomed..

[34] Amin I., Zamaliah M.M., Chin W.F. (2004). Total antioxidant activity and phenolic content in selected vegetables. Food Chem..

[35] Chang C., Yang M., Wen H., Chern J. (2002). Estimation of total flavonoid content in propolis by two complementary colorimetric methods. J. Food Drug Anal..

[36] Pechyen C., Ponsanti K., Tangnorawich B., Ngernyuang N. (2021). Waste fruit peel-mediated green synthesis of biocompatible gold nanoparticles. J. Mater. Res. Technol..

[37] Ratnayake K.R.M.S.H., Deraniyagala S.A., Udukala D. (2018). Antioxidant activity, total phenolic and total flavonoid contents of the peel and seed of *Spondias dulcis* (Amberalla) fruit. Pharma. J. Sri Lanka.

[38] Ahmad T., Bustam M.A., Irfan M., Moniruzzaman M., Asghar H.M.A., Bhattacharjee S. (2019). Mechanistic investigation of phytochemicals involved in green synthesis of gold nanoparticles using aqueous *Elaeis guineensis* leaves extract: role of phenolic compounds and flavonoids. Biotechnol. Appl. Biochem..

[39] Amendola V., Pilot R., Frasconi M., Marago O.M., Iati M.A. (2017). Surface plasmon resonance in gold nanoparticles: a review. J. Phys.: Condens. Matter.

[40] Barnes W.L., Dereux A., Ebbesen T.W. (2003). Surface plasmon subwavelength optics. Nature.

[41] Zuber A., Purdey M., Schartner E., Forbes C., van der Hoek B., Giles D., Abell A., Monro T., Ebendorff-Heidepriem H. (2016). Detection of gold nanoparticles with different sizes using absorption and fluorescence based method. Sens. Actuator B-Chem..

[42] Paul B., Bhuyan B., Purkayastha D.D. (2015). Green synthesis of gold nanoparticles using *Pogestemon Benghalensis* (B) *O. Ktz.* leaf extract and studies of their photocatalytic activity in degradation of methylene blue. Mater. Lett..

[43] Anand K., Gengan R., Phulukdaree A., Chuturgoon A. (2015). Agroforestry waste *Moringa oleifera* petals mediated green synthesis of gold nanoparticles and their anti-cancer and catalytic activity. J. Ind. Eng. Chem..

[44] Emmanuel R., Karuppiah C., Chen S.M., Palanisamy S., Prakash P. (2014). Green synthesis of gold nanoparticles for trace level detection of a hazardous pollutant (Nitrobenzene) causing Methemoglobinaemia. J. Hazard. Mater..

[45] Muthuvel A., Adavallan K., Balamurugan K., Krishnakumar N. (2014). Biosynthesis of gold nanoparticles using Solanum nigrum leaf extract and screening their free radical scavenging and antibacterial properties. Biomed. Preven. Nutr..

[46] Łojewska J., Mis ´kowiec P., Łojewski T., Proniewicz L.M. (2005). Cellulose oxidative and hydrolytic degradation: In situ FTIR approach. Polym. Degrad. Stab..

[47] Morán J.I., Alvarez V.A., Cyras V.P., Vázquez A. (2008). Extraction of cellulose and preparation of nanocellulose from sisal fibers. Cellulose.

[48] Upadhyay T.K., Fatima N., Sharma D., Saravanakumar V., Sharma R. (2017). Preparation and characterization of beta-glucan particles containing a payload of nanoembedded rifabutin for enhanced targeted delivery to macrophages. Excli. J..

[49] Keijok W.J., Pereira R.H.A., Alvarez L.A.C., Prado A.R., da Silva A.R., Ribeiro J., de Oliveira J.P., Guimarães M.C.C. (2019). Controlled biosynthesis of gold nanoparticles with *Coffea arabica* using factorial design. Sci. Rep..

[50] Jyoti K., Singh A. (2016). Green synthesis of nanostructured silver particles and their catalytic application in dye degradation. J. Genet. Eng. Biotechnol..

[51] Fernandes F.H.A., Boylan F., Salgado H.R.N. (2018). Quality standardization of herbal medicines of *Spondias dulcis* Parkinson using analytical and microbiological analysis. J. Therm. Anal. Calorim..

[52] Aljabali A.A.A., Akkam Y., Zoubi M.S.A., Al-Batayneh K.M., Al-Trad B., Alrob O.A., Alkilany A.M., Benamara M., Evans D.J. (2018). Synthesis of gold nanoparticles using leaf extract of *Ziziphus zizyphus* and their antimicrobial activity. Nanomaterials.

[53] Qu X., Yao C., Wang J., Li Z., Zhang Z. (2012). Anti-CD30-targeted gold nanoparticles for photothermal therapy of L-428 Hodgkin’s cell. Int. J. Nanomed..

[54] Khorrami S., Zarrabi A., Khaleghi M., Danaei M., Mozafari M.R. (2018). Selective cytotoxicity of green synthesized silver nanoparticles against the MCF-7 tumor cell line and their enhanced antioxidant and antimicrobial properties. Int. J. Nanomed..

[55] Saqr A.A., Khafagy E.S., Alalaiwe A., Aldawsari M.F., Alshahrani S.M., Anwer M.K., Khan S., Lila A.S.A., Arab H.H., Hegazy W.A.H. (2021). Synthesis of gold nanoparticles by using green machinery: characterization and In Vitro toxicity. Nanomaterials.

[56] Hu X., Li Z., Lin R., Shan J., Yu Q., Wang R., Liao L., Yan W., Wang Z., Shang L., Huang Y., Zhang Q., Xiong K. (2021). Guidelines for regulated cell death assays: a systematic summary, a categorical comparison, a prospective. Front. Cell Dev. Biol..

[57] Vijayakumar S., Vaseeharan B., Malaikozhundan B., Gopi N., Ekambaram P., Pachaiappan R., Velusamy P., Muruga K., Benelli G., Kumar R.S., Suriyanarayanamoorthy M. (2017). Therapeutic effects of gold nanoparticles synthesized using *Musa paradisiaca* peel extract against multiple antibiotic resistant Enterococcus faecalis biofilms and human lung cancer cells (A549). Microb. Pathog..

[58] Kamala P.M.R., Priya R.I. (2020). Antiproliferative effects on tumor cells of the synthesized gold nanoparticles against Hep2 liver cancer cell line. Egypt. Liver J..

[59] Adrie C., Bachelet M., Vayssier-Taussat M., Russo-Marie F., Bouchaert I., Adib-Conquy M., Cavaillon J.M., Pinsky M.R., Dhainaut J.F., Polla B.S. (2001). Mitochondrial membrane potential and apoptosis peripheral blood monocytes in severe human sepsis. Am. J. Respir. Crit. Care Med..

[60] Sun H., Jia J., Jiang C., Zhai S. (2018). Gold nanoparticle-induced cell death and potential applications in nanomedicine. Int. J. Mol. Sci..

[61] Han X., Jiang X., Guo L., Wang Y., Veeraraghavan V.P., Mohan S.K., Wang Z., Cao D. (2019). Anticarcinogenic potential of gold nanoparticles synthesized from *Trichosanthes kirilowii* in colon cancer cells through the induction of apoptotic pathway. Artif. Cells Nanomed. Biotechnol..

[62] Ahamed M., Akhtar M.J., Khan M.A.M., Alhadlaq H.A., Alrokayan S.A. (2015). Cytotoxic response of platinum-coated gold nanorods in human breast cancer cells at very low exposure levels. Environ. Toxicol..

[63] Li L., Zhang W., Seshadri V.D.D., Cao G. (2019). Synthesis and characterization of gold nanoparticles from Marsdenia tenacissima and its anticancer activity of liver cancer HepG2 cells. Artif. Cells Nanomed. Biotechnol..

[64] Wang L., Xu J., Yan Y., Liu H., Karunakaran T., Li F. (2019). Green synthesis of gold nanoparticles from *Scutellaria barbata* and its anticancer activity in pancreatic cancer cell (PANC‐1). Artif. Cells Nanomed. Biotechnol..

[65] Parida U.K., Biswal S.K., Bindhani B.K. (2014). Green synthesis and characterization of gold nanoparticles: study of its biological mechanism in human SUDHL-4 cell line. Adv. Biol. Chem..

